# *In silico* prediction and IgE serum screening for potential allergenicity of novel foods—a case of fungal biomass Fermotein®

**DOI:** 10.3389/falgy.2026.1746353

**Published:** 2026-05-18

**Authors:** Serge A. Versteeg, Jaap H. Akkerdaas, Lucie Pařenicová, Martine Morisset, Wieneke Dijk, Andrea O'Malley, Yvonne Dommels, Ronald van Ree

**Affiliations:** 1 Department of Experimental Immunology, Amsterdam University Medical Centers, Location AMC, Amsterdam, Netherlands; 2 The Protein Brewery B.V., Breda, Netherlands; 3 BioXact B.V., Delft, Netherlands; 4 Allergy Unit, Angers University Hospital, Angers, France; 5 Unit of Biopolymers Interactions Assemblies (UR BIA) Laboratory, INRAE, UR BIA 1268, Nantes, France; 6 Department of Biochemistry and Molecular Biology, Michigan State University, East Lansing, MI, United States

**Keywords:** allergenicity, Fermotein®, IgE cross-reactivity, ImmunoCAP, *in silico* bioinformatics, novel food, *Rhizomucor pusillus*

## Abstract

The fungus *Rhizomucor pusillus* CBS 143028 is the source of a novel food called Fermotein®, a whole fungal biomass rich in proteins, fibres, and micronutrients. To enable Fermotein® to enter the food market, the safety of the microorganism needs to be carefully assessed, including studying the allergenicity potential of proteins encoded by its genome. An *in silico* allergen database screening identified 23 proteins with more than 50% sequence identity over their full sequence, with proteins from food origin requiring allergen labelling in the European Union. These proteins are present in fish, crustaceans (e.g., shrimp, crab), peanuts, pistachio nuts, sesame seeds, and wheat. To unequivocally establish the likelihood of potential cross-reactivity of the proteins present in the novel food with those established allergens, sera of patients with doctor-diagnosed allergies to these six food categories were screened using ImmunoCAP technology. Of the 106 sera samples tested, 16 showed low IgE binding to Fermotein®. In 11 out of the 16 sera, it was demonstrated by IgE inhibition assays with Proteinase K-digested Fermotein® that all reactivity was directed to fungal carbohydrate moieties. For the remaining five sera, ImmunoCAP inhibition assays with the relevant allergen extracts and with purified human proteins highly homologous to the implicated allergen molecules demonstrated that there was no evidence to support significant cross-reactivity with the potential allergens identified in the bioinformatics *in silico* search. It is clear that IgE recognition of Fermotein® is dominated by clinically irrelevant carbohydrates. Based on these analyses, it is safe to conclude that Fermotein® presents a negligible allergenicity risk for food-allergic patients because the observed bioinformatics hits did not translate into IgE cross-reactivity.

## 1 Introduction

From the15th of May 1997, foods that do not have a history of safe consumption in the European Union (EU) have been classified as “novel foods.” Introduction of a novel food to the EU market is granted by the European Commission based on the results of a safety evaluation by the European Food Safety Authority (EFSA) following EU Regulation 2015/2283 (1). One of the requirements presented in the EFSA Guidance (2) is the provision of data on the allergenic potential of a novel food. Fermotein® is a whole fungal biomass and its allergenic potential is low based on *in silico* analysis and a literature review of the source microorganism, *Rhizomucor pusillus* CBS 143028 (3). All 23 potential allergenic proteins identified in the *in silico* study represented minor, evolutionarily highly conserved allergens of the respective foods. These included cyclophilin (peanut), manganese superoxide dismutase (MnSOD) (pistachio), maturation-like protein (sesame seed), ElF1 superfamily transcription elongation factor (wheat), triosephosphate isomerase (shrimp, crab, and wheat), pyruvate kinase (fish), glyceraldehyde-3-phosphate dehydrogenase (fish, wheat), glucose-6-phosphate isomerase (fish), and enolase (shrimp, fish). These proteins are highly conserved among many species, including humans. Based on *in silico* analysis, the risk of allergenic cross-reactivity of Fermotein® was concluded to be low (3). Nevertheless, regulatory requirements indicate that the observed *in silico* hits need to be followed up with IgE serum screening to rule out or confirm potential IgE cross-reactivity.

The FAO and the WHO have proposed the number of sera that should be tested to demonstrate—with a certainty of 95%, 99%, or 99.9%—whether a protein is a possible major (>50% of sera allergic to the source is positive) or minor (<50% of sera allergic to the source is positive) allergen (4). For minor allergens, 17 and 24 sera are required for 95% and 99% certainty, respectively. The FAO and the WHO recognize that the use of a smaller number of well-documented, high-quality sera (e.g., food challenge-proven allergy or convincing doctor's diagnosis supported by matching sensitization) may be preferable to the use of larger numbers of sera from subjects with less established proof of clinical allergy (e.g., self-reported or just sensitized without any clinical information). In clinical practice, the most widely applied technique for detection of serum IgE is the ImmunoCAP technique. The globally accepted cut-off for positivity is 0.35 kU/L of specific IgE (5–7).

The aim of the study was to establish whether any of the *in silico* detected sequence identities of Fermotein® proteins with established (minor) allergens translate into IgE cross-reactivity, using ImmunoCAP technology and well-documented sera of patients allergic to the implicated foods.

## 2 Materials and methods

### 2.1 Serum samples

Serum samples were kindly provided by the Biological Resource Centre of Angers University Hospital (France) (BB-0033-00038), following approval by the Regional Ethics Committee West II (CPP Ouest II, reference number CB 2016/09). All sera were obtained with the informed consent from patients or their caregivers, in accordance with the Declaration of Helsinki. Seventeen serum samples—the minimal number advised by the FAO and the WHO (4) for identifying even a minor allergen at 95% certainty—plus three extra sera per allergen were included in the study. For sesame only six samples were available; however, all were from patients with well-established sesame allergy. Instead of sera from wheat-allergic patients (not available), sera of grass-allergic patients were used as a surrogate. The justification for this is that there is a high degree of cross-reactivity between wheat, a cultivated grass, and wild grasses. A BlastP search at the NCBI database of the wheat allergens with >50% overall sequence identity to *R. pusillus* homologues against the allergenic *Lolium perenne* grass species showed an overall high sequence identity (88%, 93%, and 96% for Tri a 31, Tri a 45, and Tri a 34, respectively; data not shown). All patients had a convincing doctor-diagnosed history and matching IgE sensitization for one of the five relevant foods or for grass pollen, confirmed by serum IgE testing and positive skin prick-test (SPT) or prick-to-prick tests (see Supplementary Table 1). Some cases were confirmed by oral food challenge: 10/20 for peanut, 7/20 for pistachio (an additional one for cashew which is highly cross-reactive with pistachio), 3/6 for sesame, 3/20 for fish, and 1/20 for shrimp. Oral challenges could not be performed for all patients to confirm persistence of the respective allergies at the time of serum collection; however, in those tested, all patients tested positive for the food in question.

### 2.2 Preparation of Fermotein® ImmunoCAPs

Fermotein® extract was prepared using phosphate-buffered saline (PBS), essentially as described by Terlouw et al. (8). In short, Fermotein® powder (2.6 g) was mixed with 20 mL of ice-cold PBS solution in a sterile 50-mL Greiner tube. The cells were disrupted using Ultra turrax at three cycles of 10 s each. The samples were kept on ice during the disruption procedure. The samples were centrifuged at 4,000×*g* for 30 min at 4 °C. The supernatant was collected with a sterile pipet and centrifuged once more under the same conditions for 20 min to remove possible carry-over debris of the cells. Subsequently, 15 mL of the supernatant was concentrated using a Vivaspin® Turbo 15 Centrifugal Filter Device (Sartorius, 224900767; molecular cut-off value 3 kDa) to 1.046 mg/mL protein. The concentrated Fermotein® extract was stored at −20 °C until further use. Biotinylation of Fermotein® extract was performed using EZ-Link Sulfo-NHS-LC-Biotin 6 (Pierce, Rockford, IL, USA), following the manufacturer's instructions. A 10-fold molar excess of biotin was used, assuming 30 kDa as an estimate of the average molecular mass of proteins in Fermotein® extract. To check whether the biotinylated Fermotein® extract is representative of non-biotinylated Fermotein® extract, the banding pattern of non-biotinylated extract on sodium dodecyl sulfate polyacrylamide gel electrophoresis (SDS-PAGE)/Coomassie stained gel was compared to the banding pattern of the biotinylated extract. For the biotinylated extract, the banding pattern was evaluated by SDS-PAGE and subsequent immunoblotting, using streptavidin-horseradish peroxidase (HRP) for visualization. The banding patterns of the biotinylated extract were similar to those of the non-biotinylated Fermotein® extract, confirming the representativeness of the extract (Supplementary Figure 1A). Subsequently, biotinylated Fermotein® extract was coupled with streptavidin ImmunoCAPs (Ro212; Thermo Fisher Scientific, Uppsala, Sweden) at 2.5 μg/CAP, following the manufacturer's instructions.

### 2.3 Proteinase K digestion of Fermotein® extract

To digest all proteins of the Fermotein® extract, Proteinase K agarose beads were used following the manufacturer's instructions (Sigma Aldrich, Darmstadt, Germany). In short, 1.0 mL of Fermotein® extract (1.046 mg/mL protein) was incubated with 150 μL *Tritirachium album* Proteinase K immobilized on agarose beads (1 U) overnight at room temperature (RT). As a negative control, Fermotein® extract was incubated with unconjugated control agarose beads. Digestion was monitored by SDS-PAGE/Coomassie staining (Supplementary Figure 1B). Proteinase K-digested (PKd) Fermotein® extract was used in IgE ImmunoCAP inhibition assays to establish whether IgE binding to Fermotein® was directed towards carbohydrate moieties or not.

### 2.4 ImmunoCAP and ImmunoCAP inhibition

All serum samples were tested for IgE reactivity against Fermotein®, using Fermotein® ImmunoCAPs, and for IgE reactivity against implicated foods or pollen, using commercially available ImmunoCAPs (Thermo Fisher Scientific): peanut (f13), pistachio (f203), sesame (f10), grass pollen (g6), fish (f3), and shrimp (f24). Sera that were positive for IgE against Fermotein® (≥0.35 kU/L) were tested for IgE binding to cross-reactive carbohydrate determinants (CCD) using MUXF3 (xylose/fucose containing plant complex glycan) ImmunoCAPs (Thermo Fisher Scientific; o214) as a marker for IgE recognition of carbohydrate moieties. ImmunoCAP tests were carried out following the manufacturer's instructions (Thermo Fisher Scientific) with 40 μL undiluted serum per test. For ImmunoCAP inhibition, serum (70 μL) was pre-incubated with extracts (70 μL at 2 mg/mL) of food (peanut, pistachio, sesame, fish, or shrimp), timothy grass pollen, or Fermotein® (undigested and Proteinase K-digested) for 30 min at RT. Then, 40 μL of the mixture was used in ImmunoCAP testing following the standard protocol. ImmunoCAP inhibition was also carried out with purified human equivalents of cyclophilin (peanut sera), MnSOD (pistachio sera), enolase (shrimp sera), and triosephosphate isomerase (shrimp and grass pollen sera), in this case with a protein concentration of 200 μg/mL. The purified human proteins were obtained from Abcam (UK) and were expressed in *Escherichia coli*. The corresponding product numbers are ab93946 (MnSOD), ab89248 (enolase), ab86219 (cyclophilin), and ab100826 (triosephosphate isomerase). These four were chosen because proteomics analysis (personal communication, LP) showed that they had the highest relative protein abundance (1%–5%), whereas the other proteins were either not detected or present at very low levels (0.1%–0.5%).

### 2.5 Modelling surface-exposed residues

To gauge how overall sequence identity of Fermotein® protein and homologous allergen pairs translates into surface-exposed similarity, we used Alphafold 3.0 to generate pdb-files of their 3D structures (https://alphafoldserver.com/). These files were subsequently uploaded into ChimeraX (https://www.cgl.ucsf.edu/chimerax/) and pairwise assessed for surface-exposed identity.

## 3 Results

### 3.1 Structural similarity of Fermotein® protein and minor allergen pairs

**Figure 1:**
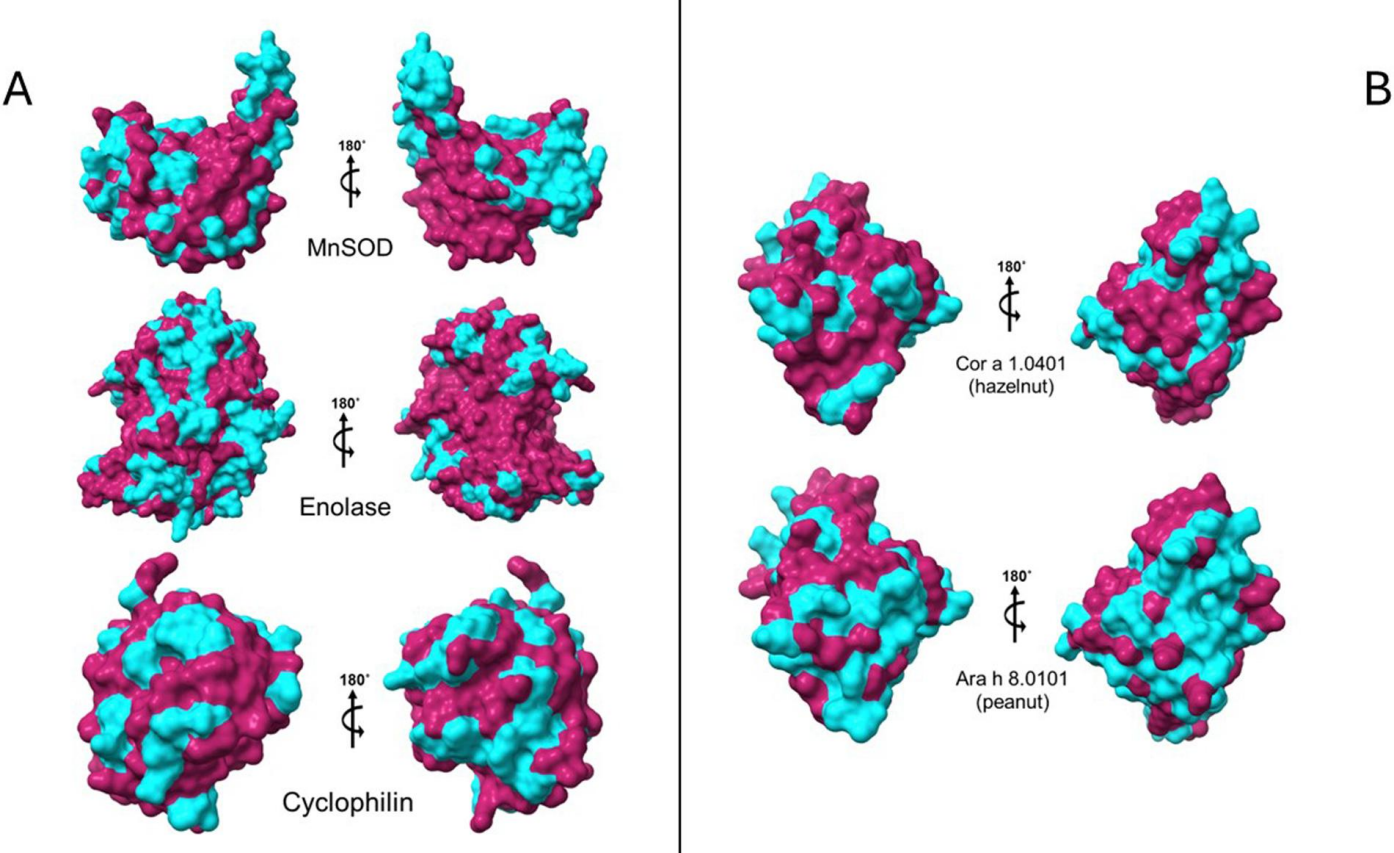
Surface-exposed similarity of Fermotein® proteins with their allergen homologues **(A)** and PR-10 food allergens with birch pollen Bet v 1 **(B)**. Identical residues are displayed in magenta, non-identical in cyan.

Bioinformatic screening of the Fermotein® genome against the Allergen Online database resulted in a series of hits with >50% full-sequence identity to minor allergens from foods and pollen, belonging to evolutionarily highly conserved protein families such as cyclophilin, MnSOD, and enolase (3). Alphafold 3.0-generated models of these Fermotein® proteins and their minor-allergen homologues confirmed high structural resemblance. ChimeraX analysis demonstrated significant similarity in patches of surface-exposed residues (Figure 1A). For comparison, we also performed the same type of analysis for established cross-reactive PR-10 allergens: birch pollen Bet v 1 (primary sensitizer), hazelnut Cor a 1, and peanut Ara h 8. Cor a 1 is establhed to be significantly more cross-reactive with Bet v 1 than Ara h 8. This is clearly reflected by a higher degree of surface-exposed identity of Cor a 1 with Bet v 1 than that of Ara h 8 (Figure 1B). Fermotein protein-allergen pairs, nevertheless had very similar degrees of surface identity as the PR-10 pairs but without proof of cross-reactivity.

### 3.2 IgE against Fermotein®

**Figure 2:**
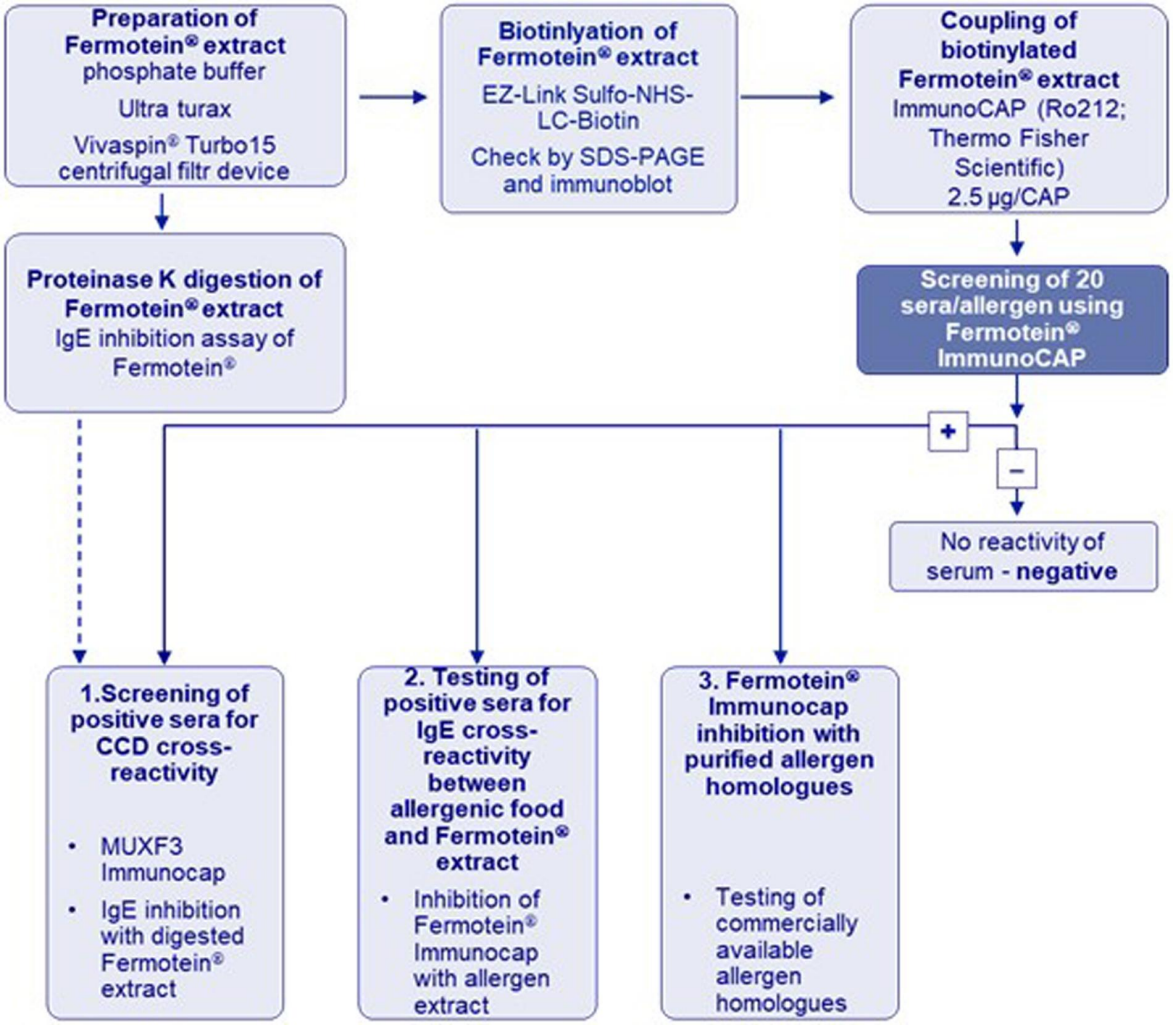
Step-by-step evidence-based approach to evaluate potential IgE cross-reactivity of Fermotein® proteins to known allergens.

Serum samples from patients with established allergy to the implicated foods and pollen (Supplementary Table 1) were used to determine whether evidence could be found for cross-reactivity to Fermotein® of IgE in sera of allergic patients to peanut, fish, shrimp, pistachio, wheat (grass pollen), and sesame. This was done in a stepwise manner (Figure 2).

In the first step, IgE recognition of Fermotein® extract was assessed. A total of 106 sera of patients with convincing doctor-diagnosed allergies (20 per implicated food, except 6 for sesame) were screened for IgE against Fermotein® using ImmunoCAP. Only sixteen sera were positive (≥0.35 kU/L, Supplementary Table 1). The highest IgE response was 8.62 kU/L (serum of patient Pe#1), but most IgE titres were low (between 0.35 and 2.00 kU/L in 13 out of 16 cases).

### 3.3 Role of carbohydrate in IgE recognition of Fermotein®

**Figure 3:**
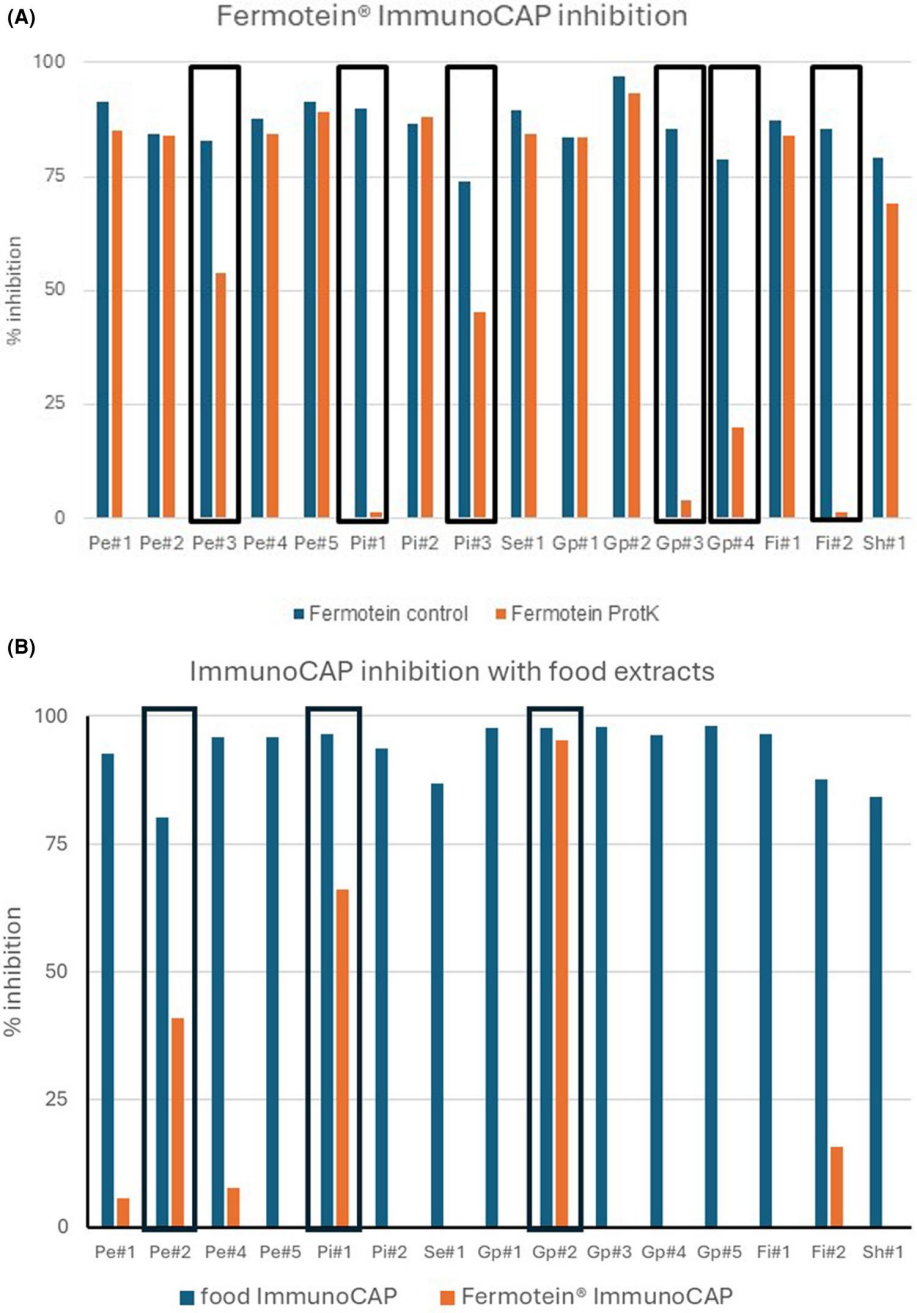
ImmunoCAP inhibitions. **(A)** Fermotein® ImmunoCAP inhibition with proteinase K (un)digested Fermotein® extract. Sera partially inhibited by proteinase K-digested Fermotein® are shown in black box (*n* = 6). **(B)** ImmunoCAP inhibition with food/pollen extracts. Inhibition of binding to food/pollen ImmunoCAPs is represented by blue bars and to Fermotein® by red bars. Sera significantly (>40%) inhibited by food extract are in black box (*n* = 3). (The serum Pe#3 was not available for this experiment). In both experiments, the percent inhibition was calculated relative to an uninhibited control (PBS buffer instead of extract).

CCDs are carbohydrate moieties of glycoproteins that can induce the production of highly cross-reactive IgE antibodies. To investigate carbohydrate involvement in the IgE recognition of Fermotein®, the 16 positive sera were first screened for IgE binding to plant-derived CCDs, generally acknowledged as clinically irrelevant (9). Seven out of 16 sera were positive (Supplementary Table 1). To further investigate the possible role of carbohydrate-based IgE binding, Fermotein® ImmunoCAP inhibition assays were performed with PKd Fermotein®. Ten sera were fully inhibited, but 6 out of 16 sera were only partially inhibited or not inhibited (Figure 3A). The 10 sera that were fully inhibited included the 7 plant-derived CCD-positive sera (Supplementary Table 1). The other three that were fully inhibited by PKd Fermotein® likely recognized more specific fungal carbohydrates.

### 3.4 Inhibition of IgE binding to Fermotein® with allergen extract

To investigate whether IgE cross-reactivity occurred with foods from which the *in silico-*identified food allergen hits originate, the 16 Fermotein® positive sera were tested by Fermotein ImmunoCAP inhibition using the respective food extracts as inhibitor (Figure 3B). While for most sera little or no inhibition of IgE binding to Fermotein® was observed, in three cases (serum Pe#2, Pi#1, and Gp#2) significant inhibition (>40%) was detected. For two of these sera—Pe#2 and Gp#2—inhibition could be explained by carbohydrate-based IgE recognition and not protein-based cross-reactivity. However, for pistachio serum Pi#1, involvement of a cross-reactive protein was implicated because PKd Fermotein® extract did not inhibit IgE binding (Figure 3A).

### 3.5 Inhibition of IgE binding to Fermotein® with human homologues of predicted food allergens

To further investigate potential cross-reactivity to a protein in Fermotein® with homology to the *in silico* predicted food allergens, ImmunoCAP inhibitions were performed with commercially available human homologues of four respective minor allergens—cyclophilin, MnSOD, triosephosphate isomerase and enolase (see Section 2.4). IgE binding to Fermotein® based on protein interaction was only seen for the pistachio extract. No inhibition of IgE binding to Fermotein® was observed with the MnSOD human homologue for pistachio sera Pi#1, Pi#2, and Pi#3, demonstrating that the only observed probable protein-mediated cross-reactivity (Pi#1) was not caused by MnSOD. Furthermore, no inhibitions were observed with cyclophilin for peanut sera (Pe#1, Pe#3, and Pe#5), triosephosphate isomerase for shrimp (Sh#1) and grass pollen (Gp#2), or enolase for shrimp (Sh#1) (data not shown).

## 4 Discussion

For establishing the relevance of positive hits found in the bioinformatics *in silico* search of Fermotein® against the AllergenOnline database (3), a serum screen was performed for six of the implicated allergenic food sources. For each of the allergen sources, 20 sera from patients with established allergy (confirmed by SPT and/or oral food challenge) were used, except for sesame, where only six sera were available. For wheat, sera from grass pollen allergic patients were used as surrogate because of the scarcity of sera from wheat-allergic patients. The identified allergens in wheat with sequence similarity—ElF1 transcriptions factor (Tri a 45), triosephosphate isomerase (Tri 31), and glyceraldehyde 3P dehydrogenase (Tri 34)—were reported to be respiratory allergens in baker's asthma (10–12). Although occupational exposure to wheat flour is quite different from normal environmental exposure to pollen, without access to sera from patients with baker's asthma, the use of sera from grass pollen allergic patients is a justifiable alternative. Proteins homologous to all three wheat allergens are found in common temperate-climate allergenic grasses such as *L. perenne*, with high degrees of identity with their wheat counterparts.

Out of 106 sera screened, 16 showed IgE reactivity against Fermotein®. Only five sera were not (completely) inhibited by PKd Fermotein® (Figure 3A), excluding the involvement of carbohydrates. These sera—Pi#1, Pi#3, Pe#7, Gp#3, Gp#4, and Fi#2—showed IgE recognition of a protein in Fermotein®. However, IgE recognition was very low in all five cases: 1.00, 0.36, 0.58, 0.36, and 0.56 kU/mL, respectively (Supplementary Table 1). To establish whether such IgE cross-reactivity exists, Fermotein® ImmunoCAP inhibition assays were performed with extracts of the implicated foods or pollen for all sera that responded to Fermotein® with values >0.35 kU/L. As controls, the relevant food or pollen ImmunoCAP was also inhibited with the food and pollen extracts tested (homologous inhibitions). For peanut, there was not enough serum Pe#3 (the lowest, 1.1 kU/L, of the five positives on Fermotein®) left to do the homologous control inhibition analysis (peanut ImmunoCAP with peanut). For the four remaining peanut sera, homologous inhibitions were all complete (Figure 3B). Peanut extract, however, could not inhibit IgE binding to Fermotein® in three out of these four cases (Figure 3B), demonstrating that the IgE reactivity of the specific patient's serum is not related to cross-reactivity with peanut. For Pe#3, no inhibition was observed (not shown). It can of course not be fully excluded that there is not enough of the targeted cross-reactive protein (cyclophilin) present in the peanut extract to inhibit IgE binding. Only serum Pe#2 showed some degree of inhibition (Figure 3B). However, for this serum, IgE binding to Fermotein® was fully inhibited by digested Fermotein® extract, suggesting that the most likely explanation for the partial inhibition with peanut extract is peanut CCD rather than a protein (Figure 3A). For pistachio, homologous pistachio inhibitions were complete (Figure 3B) in the two sera tested (Pi#1 and Pi#2). For one of those (Pi#1), the IgE binding to Fermotein® was significantly inhibited by pistachio extract (Figure 3B), but not by PKd Fermotein® extract (Figure 3A), suggesting involvement of a cross-reactive protein in pistachio extract, possibly MnSOD, a protein identified in the *in silico* analysis (3). For the sesame positive serum, homologous inhibition was complete, but no inhibition was observed for IgE binding to Fermotein® with sesame extract (Figure 3B). This indicates that there is no support for cross-reactivity between Fermotein® and a protein allergen in sesame. For grass pollen, homologous inhibitions were complete for all five sera tested (Figure 3B). IgE binding to Fermotein® could not be inhibited in four out of five cases (Figure 3B). Only for serum Gp#2, inhibition of IgE binding to Fermotein® with grass pollen extract was complete (Figure 3B). Since IgE binding to Fermotein® was also completely inhibited with digested Fermotein® extract (Figure 3A), it is most likely that grass pollen CCD is at the basis of the inhibition observed. For sera Gp#3 and Gp#4, almost no inhibition was observed with PKd Fermotein® extract (Figure 3A), suggesting the involvement of a protein. In the absence of cross-reactivity with grass pollen, the borderline IgE recognition (0.58 and 0.36 kU/L, respectively) is unlikely to be related to wheat allergens, although it cannot be ruled out completely that a potential cross-reactive wheat allergen is underrepresented in the grass pollen extract used as surrogate. Homologous inhibitions for fish were complete, but IgE binding to Fermotein® could not be inhibited by fish extract (Figure 3B), suggesting that there is no cross-reactivity between fish and Fermotein®. Similar results were found for the shrimp extract and serum Sh#1 (Figure 3B). Overall, there is no convincing evidence to support significant IgE cross-reactivity between Fermotein® and the *in silico-*identified (minor) allergenic food or pollen proteins. The minor degree of cross-reactivity that was found was in most cases carbohydrate-driven rather than protein-driven, except for probably one serum from a pistachio-allergic patient (Pi#1).

To further exclude potential cross-reactivity to a protein in Fermotein®, given that several of the predicted minor cross-reactive allergens are likely to be present in only small quantities in the protein extracts, ImmunoCAP inhibitions were performed with commercially available purified human homologues of the respective minor allergens from peanut, pistachio, fish, wheat/grass pollen, and shrimp: Human cyclophilin (for peanut), MnSOD (for pistachio), enolase (for shrimp), and triosephosphate isomerase (for fish and wheat/grass pollen) were used as surrogate inhibitors. Purified versions of these minor allergens from the respective foods (or from Fermotein®) would have been better suited, but these were not available. The justification for using human homologues is that they share similar levels of sequence identity to the homologous proteins in Fermotein® as those observed for the food allergens identified in the bioinformatics screening of the allergen database [Table 1, (3)]. These protein families are simply too conserved in evolution to be likely candidates for significant (IgE) immune responses. ImmunoCAP inhibitions were not performed with the other proteins with sequence homology to proteins present in food requiring allergen labelling as these proteins were not present or only at very low abundancy in Fermotein®, based on proteomics experiments (data not shown). None of the purified proteins tested could inhibit IgE binding to Fermotein® extract. This was particularly important for serum Pi#1, in which IgE binding to Fermotein® was inhibited by pistachio but not by PKd Fermotein®. The lack of inhibition with human MnSOD demonstrates that it is highly unlikely that this enzyme in Fermotein® is involved in IgE cross-reactivity with pistachio nuts. No other proteins in Fermotein® were identified in the bioinformatics screening as homologous to other allergens in pistachio nuts.

**Table 1:** Percent of full sequence identity of the *R. pusillus* proteins to the corresponding identified allergen and to the homologous human proteins (see Section 2.4).

*R. pusillus* protein (gene code)	Protein function	IUIS allergen name; accession no.	Species	% identity to allergen	% identity to human protein
scf001g0241	Enolase	Pan h 2; XP_026777250.1	*Pangasianodon hypophthalmus* (striped catfish)	74.90	71
scf028g0035	Enolase	no IUIS name; AEM89226.1	*Fenneropenaeus merguiensis* (banana shrimp)	61.50	72
scf028g0035	Enolase	Sal s 2; ACH70931.1	*Salmo salar* (Atlantic salmon)	74.50	72
scf044g0029	Triose-phophate isomerase	Sal s 8; ACM09737.1	*Salmo salar* (Atlantic salmon)	57.10	56
scf023g0237	MnSOD	Pis v 4; ABR29644.1	*Pistacia vera* (pistachio nut)	53.80	60
scf007g0324	Cyclophilin	Ara h 18; XP_025675300.1	*Arachis hypogaea* (peanut)	68.70	70

IUIS, International Union of Immunological Societies.

Interestingly, despite a comparable degree of surface-exposed similarity observed among established cross-reactive PR-10 allergens (Figure 1), no cross-reactivity could be demonstrated between the Fermotein® proteins and their allergen homologues. The allergens are without exception very minor, with very little supporting evidence for clinical relevance, making the chance of finding cross-reactivity with patient sera highly unlikely. Moreover, epitopes on these minor allergens do not necessarily localize in the surface-exposed areas of similarity observed in Fermotein® proteins. Most importantly, the very high identity to human homologues implies that the immune system is prone to ignore them.

## 5 Conclusion

In conclusion, twenty sera of allergic patients per food category (six for sesame) were screened for IgE cross-reactivity with Fermotein® using a stepwise, evidence-based approach. This allowed us to establish or eliminate involvement of carbohydrates, protein(s) from the respective food extract, and the *in silico*-identified target protein homologues. It can be concluded that for most of the 16 positive hits the IgE reactivity with Fermotein® was very low (0.35 and 2.00 kU/L in 13 out of 16 sera) and was carbohydrate-mediated. Only in the case of one serum for pistachio (Pi#1) was the IgE interaction clearly protein-based, but this was not related to the *in silico-*identified allergen—MnSOD. This case illustrates that the Codex weight-of-evidence approach (13) designed for genetically modified crops is not optimally fit for purpose for allergenicity assessment of novel foods containing hundreds or thousands of proteins. It results in identification of evolutionarily highly conserved proteins among many different species, including humans, that are without exception minor allergens of poorly established clinical relevance in the implicated foods, and that are very likely of negligible if any clinical relevance in novel foods such as in this case Fermotein®. For the screening of whole genomes of novel foods, it would be helpful if allergens listed in allergen databases like Allergen Online and COMPARE are graded according to their proven clinical relevance (including prevalence) and their identity to human proteins.

## Data Availability

The original contributions presented in the study are included in the article/Supplementary Material; further inquiries can be directed to the corresponding author.
