# Supplementary material for: In silico prediction and IgE serum screening for potential allergenicity of novel foods—a case of fungal biomass Fermotein®
Source: Front Allergy. 2026 May 18;7:1746353. doi: 10.3389/falgy.2026.1746353 (PMC13223168; doi:10.3389/falgy.2026.1746353)

**Supplementary Figures and Tables**

**Supplementary Table 1.** List of sera of allergic patients (for sesame allergy only 6 sera were available and instead of sera of wheat allergic patients, sera of grass allergic patients were used to surrogate). IgE screening results against Fermotein^®^ and cross-reactive carbohydrates determinants MUX3 are given in the respective right columns. Positive results are in bold. (NEG means < 0.35 kU/L^3^, NT means not tested).

**Supplementary Figure 1. a.** Biotinylated Fermotein^®^ extract – visual control. Right – SDS-PAGE gel, left – Immunoblot with streptavidin. Left lane: molecular markers. **b.** Proteinase K digested Fermotein^®^ extract – Coomassie blue staining. Lane 1: undigested, Lane 2: digested, Lane 3: control agarose beads + undigested Fermotein^®^ extract.


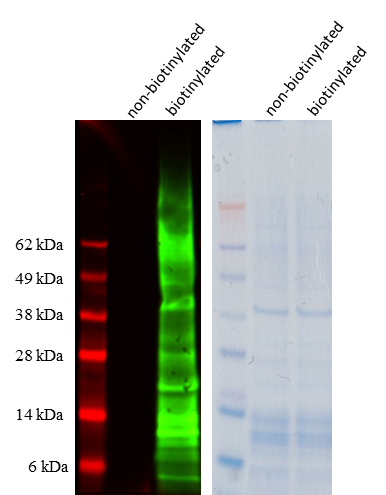
**a. b.**


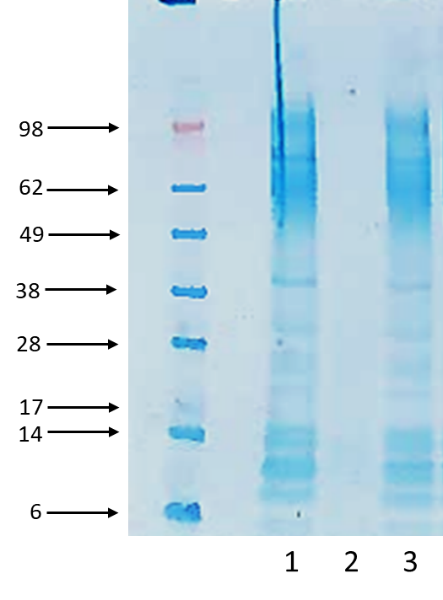

Supplement: Supplementary file 1 [file Table1.docx]
